# Adoptive B cell therapy for chronic viral infection

**DOI:** 10.3389/fimmu.2022.908707

**Published:** 2022-07-26

**Authors:** Young Rock Chung, Tanushree Dangi, Nicole Palacio, Sarah Sanchez, Pablo Penaloza-MacMaster

**Affiliations:** Department of Microbiology-Immunology, Feinberg School of Medicine, Northwestern University, Chicago, IL, United States

**Keywords:** B cells, chronic viral infection, adoptive cell therapy, lymphocytic choriomeningitis virus (LCMV), virus

## Abstract

T cell-based therapies have been widely explored for the treatment of cancer and chronic infection, but B cell-based therapies have remained largely unexplored. To study the effect of B cell therapy, we adoptively transferred virus-specific B cells into mice that were chronically infected with lymphocytic choriomeningitis virus (LCMV). Adoptive transfer of virus-specific B cells resulted in increase in antibody titers and reduction of viral loads. Importantly, the efficacy of B cell therapy was partly dependent on antibody effector functions, and was improved by co-transferring virus-specific CD4 T cells. These findings provide a proof-of-concept that adoptive B cell therapy can be effective for the treatment of chronic infections, but provision of virus-specific CD4 T cells may be critical for optimal virus neutralization.

## Introduction

T cell-based therapies, such as immune checkpoint blockade and chimeric antigen receptor (CAR) T cells, have been studied extensively. However, therapies to improve B cell responses have remained understudied. B cells can provide a long-term source of antibody, but they can also be deleted during persistent infection. Prior studies have shown that adoptive transfer of B cells very early after chronic viral infection results in limited antiviral effects, due in part to excessive inflammation that compromises the survival of B cells during the acute phase of infection (1–3). However, the effect of virus-specific B cells during the late or “chronic” stages of chronic viral infection are still not well understood.

In the present study, we evaluated whether adoptive transfer of virus-specific B cells can improve viral control during an established lymphocytic choriomeningitis virus (LCMV) infection in mice. We show that adoptive B cell transfer can improve viral control, and the efficacy of this therapy is potentiated following co-transfer of virus-specific CD4 T cells.

## Results

### Adoptive B cell therapy during an established chronic viral infection improves antibody responses

**Figure 1:**
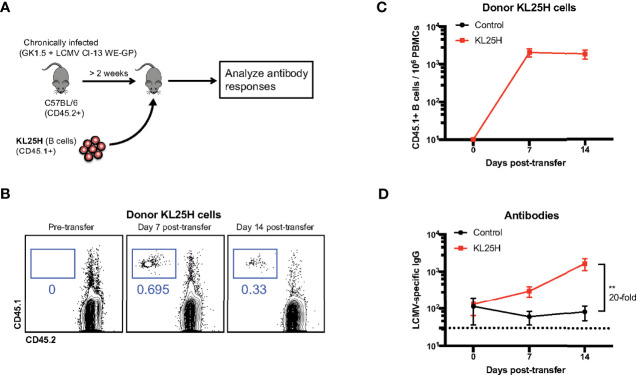
Transfer of virus-specific B cells improves antibody levels in chronically infected mice. **(A)** Experimental outline for evaluating the effect of B cell transfer during a chronic viral infection. Mice chronically infected with LCMV Cl-13 WE-GP received 5x10^6^ KL25H B cells. **(B)** Representative FACS plots showing the frequencies of KL25 B cells in blood. **(C)** Summary of KL25H B cells in blood. CD45.1 expression was used to distinguish donor cells. **(D)** Summary of virus-specific antibody levels in sera by enzyme-linked immunosorbent assay (ELISA). Experiments were repeated 5 times, n=3-5 mice per group per experiment. Error bars represent SEM. The p-values were calculated using Mann-Whitney test. **P<0.005. Dotted line represents limit of detection.

To evaluate the effect of adoptive B cell therapy, we utilized a murine model of chronic lymphocytic choriomeningitis virus (LCMV) infection (see Materials and Methods). In these experiments, we used mice that were infected with a chimeric LCMV Cl-13 virus expressing the LCMV WE virus glycoprotein (GP), also known as LCMV Cl-13 WE-GP. We utilized this chimeric virus because it is safer than parental WE virus, and because this virus is recognized by the KL25H B cells, whereas parental LCMV Cl-13 is not recognized by the KL25H B cells (4). We intravenously transferred 5x10^6^ transgenic LCMV-specific B cells (KL25H B cells) expressing the CD45.1+ congenic marker, into CD45.2+ mice that were chronically infected with LCMV Cl-13 WE-GP (**Figure 1A**). The expression of distinct congenic markers by donor and recipient cells allowed us to distinguish donor cells in circulation (**Figures 1B, C**). Importantly, transfer of B cells into chronically infected mice resulted in a 20-fold increase in antibody titers (**Figure 1D**).

**Figure 2:**
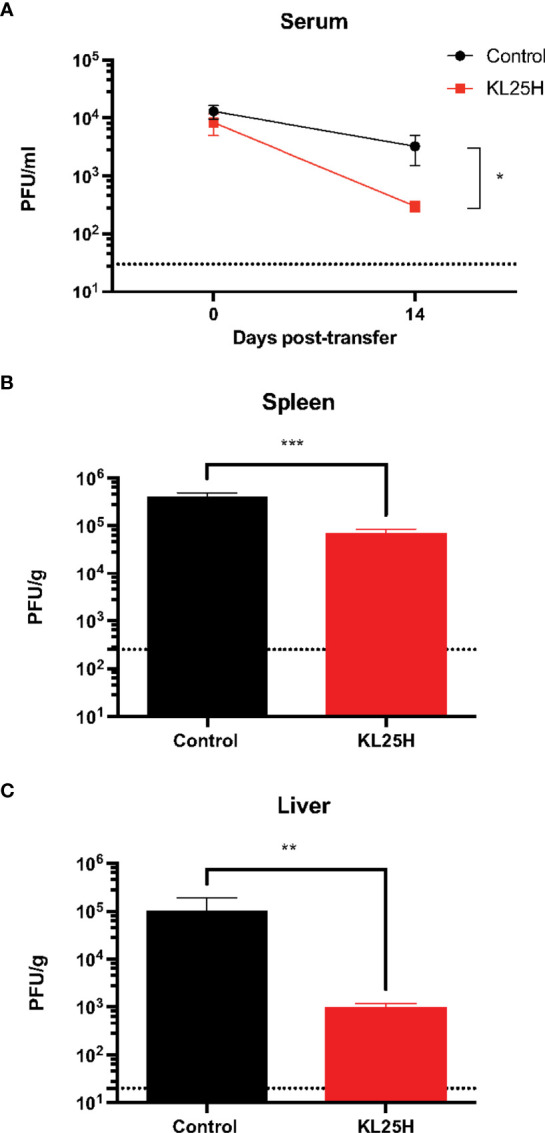
Adoptive B cell therapy improves viral control. **(A)** Summary of viral control in sera. **(B)** Summary of viral loads in spleen. **(C)** Summary of viral loads in liver. Experimental layout was similar as the one depicted in **Figure 1A**. The limit of detection is indicated by a dashed line. Experiments were performed 3 times, n=2-5 mice per experiment. Error bars represent SEM. The p-values were calculated using Mann-Whitney test. *P<0.05, **P<0.005, ***P<0.0005. Dotted line represents limit of detection.

**Figure 3:**
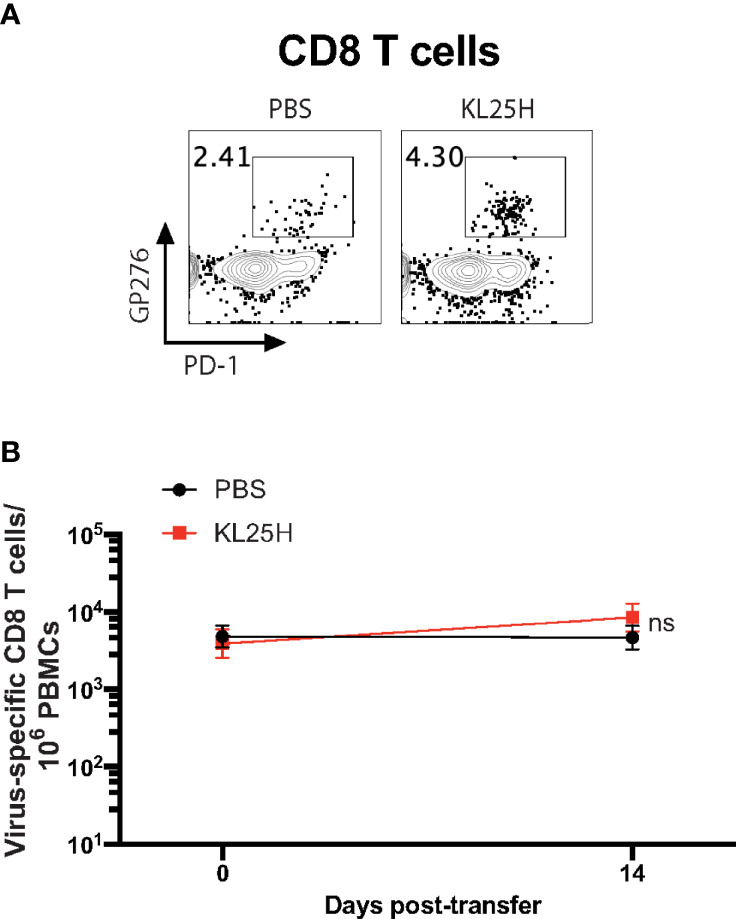
CD8 T cell responses are not significantly improved by adoptive B cell therapy. **(A)** Representative FACS plots showing the frequencies of LCMV-specific CD8 T cells in PBMCs at day 14 post-transfer. **(B)** Summary of LCMV-specific (GP276-specific) CD8 T cells. Experiments were performed 3 times, n=3-5 mice per experiment. Error bars represent SEM. The p-values were calculated using Mann-Whitney test. ns, not significant.

We then evaluated whether the B cell transfer elicited a virological effect. Transfer of virus-specific B cells into chronically infected mice resulted in enhancement in viral control (**Figure 2**). There was a pattern of ~2-fold increased CD8 T cell responses after B cell transfer, but it was not statistically significant (**Figure 3**). Altogether, these data show that transfer of virus-specific B cells can help clear a chronic viral infection.

### Antigen-specificity is required following adoptive B cell therapy

**Figure 4:**
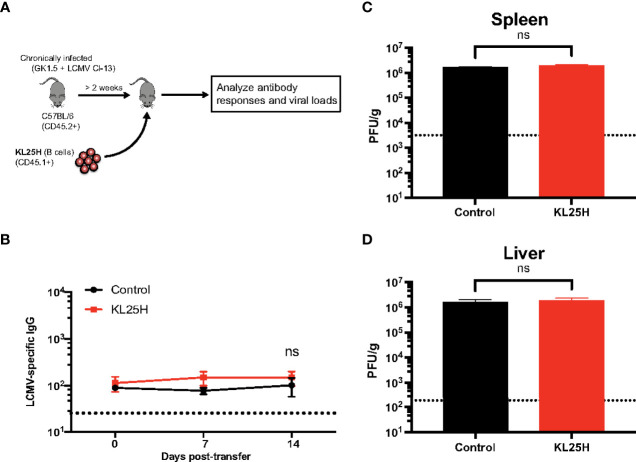
Antigen specificity is required for adoptive B cell therapy to be effective. **(A)** Experimental outline for evaluating the effect of B cell transfer during a chronic viral infection. Mice chronically infected with LCMV Cl-13 received 5x10^6^ KL25H B cells. **(B)** Summary of virus-specific antibody levels in sera. **(C)** Summary of viral loads in spleen. **(D)** Summary of viral loads in liver. The limit of detection is indicated by a dashed line. Experiments were performed 2 times, n=3-4 mice per experiment. Error bars represent SEM. The p-values were calculated using Mann-Whitney test. ns, not significant. Dotted line represents limit of detection.

B cells are not only important for humoral responses but they can also play roles in antigen presentation and regulation of adaptive immune responses (5, 6). In the experiments above, we did not ascertain whether the improvement of viral control by adoptive B cell therapy could be due other immune mechanisms that did not involve virus-specific antibodies. Therefore, to evaluate the need for “virus-specificity,” we transferred KL25H B cells into mice chronically infected with a variant virus, LCMV Cl-13 (expressing Cl-13 GP instead of the WE GP), which is not recognized by KL25H B cells (**Figure 4A**). As expected, adoptive B cell therapy did not increase antibody responses (**Figure 4B**). Moreover, there were no changes in viral loads (**Figures 4C, D**), suggesting that for B cell therapy to be effective, the donor B cell population must be highly specific for the chronic viral antigen. Altogether, adoptive transfer of B cells of irrelevant specificity does not improve viral control, suggesting that B cell therapy exerts its antiviral effect *via* virus-specific antibodies, and not *via* other mechanisms that are used by B cells to regulate adaptive immune responses.

**Figure 5:**
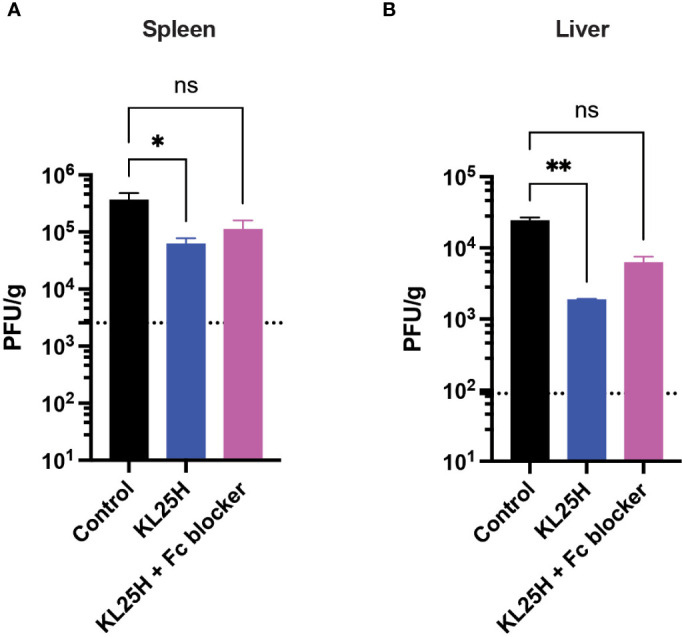
The efficacy of adoptive B cell therapy is partly dependent on antibody effector functions. An Fc receptor blocking antibody (2.4G2) was administered intraperitoneally every three days, five times (500 μg/dose). **(A)** Summary of viral loads in spleen. **(B)** Summary of viral loads in liver. Experiments were performed 2 times, n=3-5 mice per experiment. Error bars represent SEM. The P values were calculated using the Kruskal-Wallis non-parametric ANOVA test. ns, not significant. *P<0.05, ***P<0.0005, ****P<0.0001. Dotted line represents limit of detection.

We also interrogated the mechanism by which adoptive B cell therapy improved viral control, and we evaluated whether blocking antibody-dependent cellular cytotoxicity (ADCC) using an Fc receptor blocker (7) could abrogate the antiviral effect of adoptive B cell therapy. Fc receptor blockade reduced the efficacy of adoptive B cell therapy, suggesting that antibody effector functions were partially (but not solely) involved in the antiviral effect (**Figures 5A, B**). Taken together, these data show that adoptive transfer of virus-specific B cells improves the control of a chronic LCMV infection due in part to antibody effector functions.

### Synergistic effects of B Cell and CD4 T cell therapy

**Figure 6:**
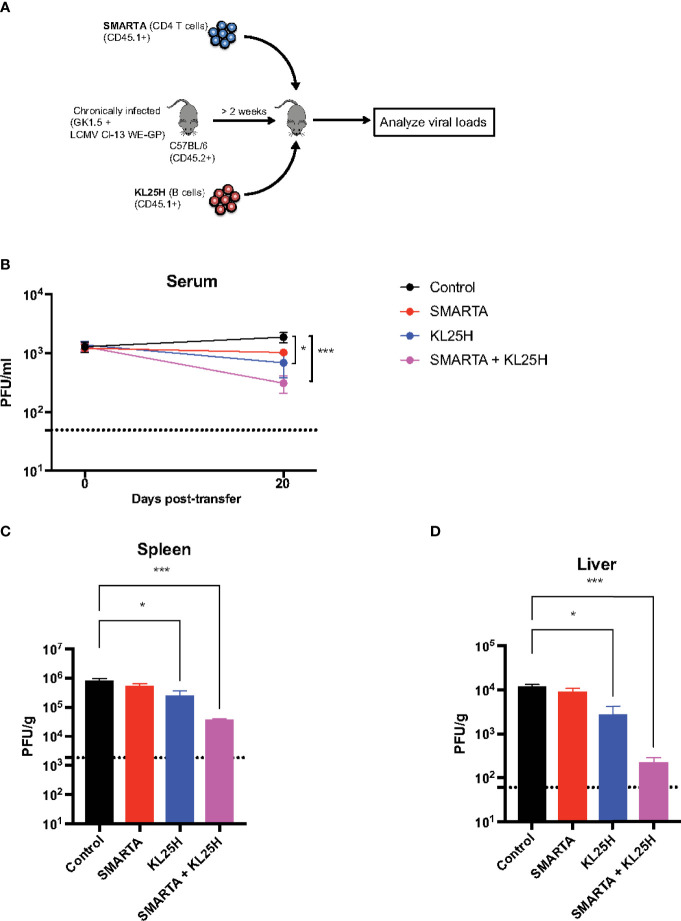
Virus-specific CD4 T cells enhance adoptive B cell therapy. **(A)** Experimental outline for evaluating whether LCMV-specific CD4 T cells augment the efficacy of adoptive B cell therapy. **(B)** Summary of viral loads in serum. **(C)** Summary of viral loads in spleen. **(D)** Summary of viral loads in liver. Experiments were performed 2 times, n=3-5 mice per experiment. Error bars represent SEM. The P values were calculated using the Kruskal-Wallis non-parametric ANOVA test. ns, not significant. *P<0.05, ***P<0.0005. Dotted line represents limit of detection.

CD4 T cells play a critical role in helping B cell responses. Therefore, we co-transferred LCMV-specific CD4 T cells (SMARTA) along with KL25H B cells to determine if this combined cell transfer regimen resulted in a synergistic improvement in viral control (**Figure 6A**). Mice that received both CD4 T cells and B cells exhibited a more significant reduction in viral loads, compared to mice that received B cells alone (**Figures 6B–D**).

**Figure 7:**
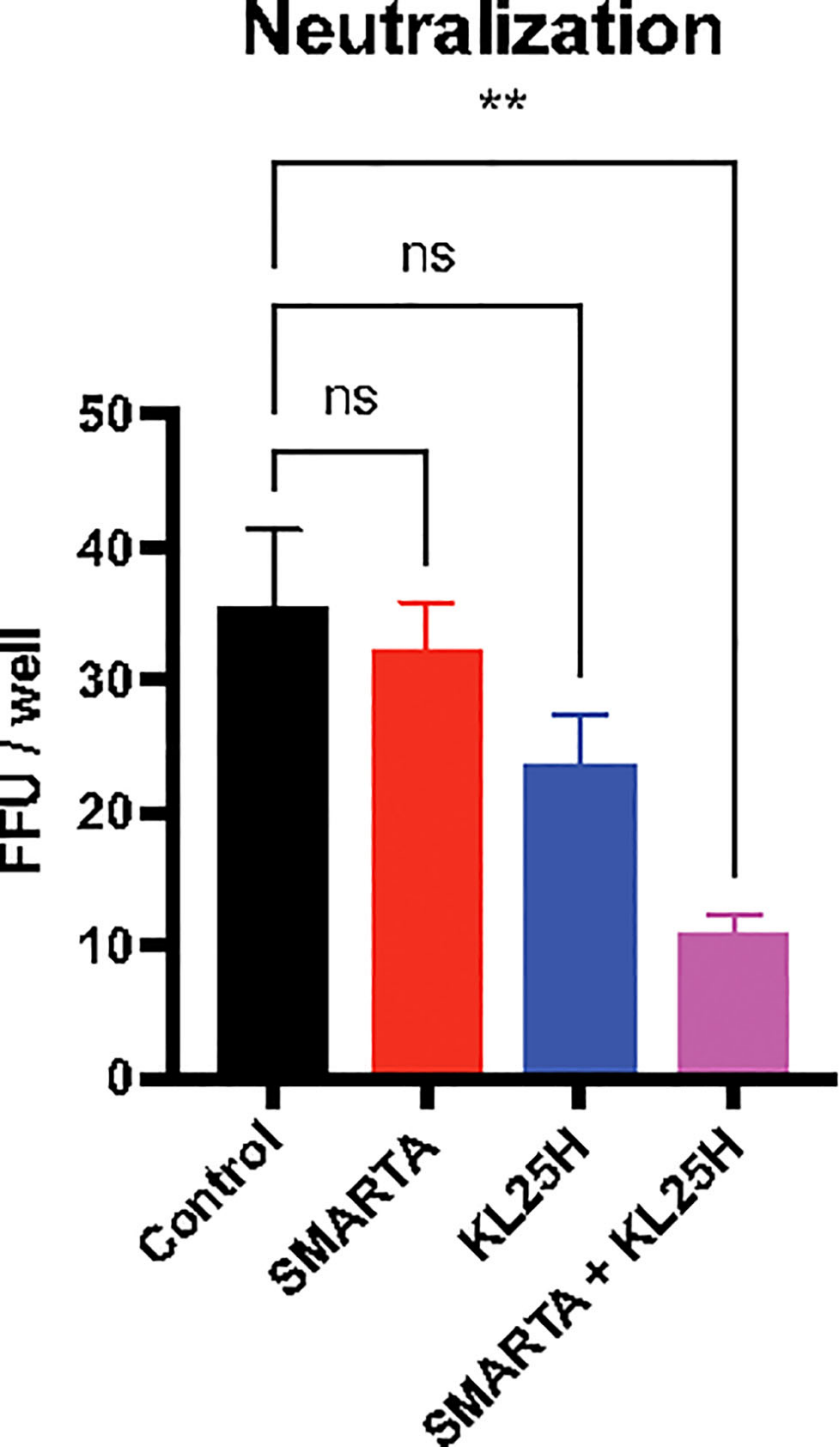
Combined therapy with CD4 T cells and B cells enhances neutralizing antibody responses. Summary of neutralization assays, using 100 PFU of LCMV Cl-13 WE-GP virus, cultured with mouse sera (day 14 post-transfer). Sera were diluted 40-fold. Two days after infection, monolayers were stained with a fluorescently labeled anti-LCMV antibody (VL4) and results were plotted as focus forming units (FFU). Experiments were performed 2 times, n=3-5 mice per experiment. Error bars represent SEM. The P values were calculated using the Kruskal-Wallis non-parametric ANOVA test. ns, not significant, **P<0.005. Dotted line represents limit of detection.

Antibodies exert antiviral functions *via* various mechanisms, including antibody effector functions as discussed earlier, as well as antibody neutralization (8). We interrogated whether adoptive B cell therapy improved antibody neutralization, and whether CD4 T cells, known to exert helper functions (9–13), could enhance this type of antibody function. Adoptive B cell therapy resulted in a pattern of improved antibody neutralization, but this was not statistically significant relative to control (**Figure 7**). However, co-transfer of SMARTA CD4 T cells and KL25H B cells resulted in a statistically significant improvement in antibody neutralization, suggesting that helper CD4 T cells are critical for inducing neutralizing antibody responses by the adoptive B cell therapy (**Figure 7**). This improvement in neutralization by provision of CD4 T cells was associated with a pattern of increased frequencies of isotype-switched B cells and germinal center (GC) B cells in the spleen, but the differences were not statistically significant (p=0.06 for isotype-switched B cells, **Figure S1**). Co-transfer of CD4 T cells and B cells did not improve CD8 T cells (**Figure S2**).

All of the experiments above utilized a model of chronic infection induced by experimental CD4 T cell depletion at the time of challenge, because without experimental CD4 T cell ablation it is not possible to induce a stringent lifelong multi-organ infection (11). However, we also utilized a model of protracted infection caused by transient IFN-I blockade at the time of challenge (**Figure S3A**). In this protracted infection model, which did not involve experimental CD4 T cell ablation, we also observed reduction of viral loads after B cell therapy (**Figure S3B**).

In conclusion, we utilized a model of chronic viral infection to evaluate the efficacy of adoptive B cell therapy, and we demonstrate that it can help control a persistent viral infection, especially if combined with CD4 T cell therapy.

## Discussion

Chronic infections afflict millions of people worldwide. In this study, we evaluated the effect of adoptive B cell therapy during chronic viral infection in mice. We adoptively transferred B cells that express the heavy chain of an LCMV-specific B cell receptor (BCR), and we showed that this resulted in decrease in viral loads. Recently, Voss et al. developed a technique to knock in the BCR heavy chain of an HIV-specific antibody into human B cells *in vitro* (14). Upon activation, antibodies secreted by these B cells exhibited neutralization capacity (14). Although that prior study was performed *in vitro*, it suggests the feasibility of “CAR B cell therapy” for the treatment of chronic viral infection, especially by modifying the host’s B cell specificity *ex vivo* prior to infusion.

Memory antibody responses play a critical role in preventing viral infections, especially by neutralizing the virus before it enters host cells. Once the infection occurs, T cells are critical for the clearance of virally-infected cells and control the infection (11, 15–17). The role for B cells in controlling a persistent viral infection, however, is still unclear. A prior study showed that B cell depletion in an HIV-infected individual who had a B cell malignancy resulted in increase in HIV viremia (18). Another study showed that transfer of immune splenocytes (containing memory B cells and CD4 T cells) into neonatally infected mice results in clearance of infection (19), also suggesting that B cells may help clear chronic viral infection, although in that study the adoptive transfers involved highly polyclonal populations of memory lymphocytes harvested from previously infected mice.

Due to biosafety reasons, we did not utilize wild type LCMV WE in our laboratory. Instead, we used a chimeric LCMV Cl-13 expressing the WE glycoprotein (GP), which is recognized by KL25H B cells. This chimeric LCMV Cl-13 WE GP virus is highly attenuated relative to the parental Cl-13 or WE, and in our hands, chronic infection could only be induced by depleting CD4 T cells at the time of challenge. Thus, we utilized the CD4 T cell depleted model used in previous reports (11, 20, 21). Our results highlight that helper CD4 T cells are critical for B cell therapy, especially because they help to improve neutralizing antibody responses. CD4 T cells provide critical signals that improve B cell functions (12, 22–26), which explains the synergy of combined adoptive cell therapy. Taken together, we show that adoptive B cell therapy can improve the control of a chronic viral infection. These data warrant the clinical evaluation of adoptive B cell therapies for the treatment of chronic viral infection.

## Materials and methods

### Mice, infections and treatments

6-8-week-old female and male C57BL/6 mice from Jackson laboratories were used as recipients in all experiments. All infections were intravenous (i.v.) *via* the lateral tail vein and using a mouse restrainer. All antibodies for *in vivo* treatments were purchased from BioXCell, and were diluted in sterile PBS. For generating chronic viral infections, mice were infected with 2x10^6^ plaque forming units (PFU) of LCMV Cl-13 expressing the LCMV WE glycoprotein (GP), referred to as LCMV Cl-13 WE-GP. 500 μg of a CD4 depleting antibody (GK1.5) was administered intraperitoneally at the time of infection to induce chronic multi-organ infection as described previously (11). In another model of infection, 500 μg of an IFNAR1 blocking antibody (MAR1-5A3) was administered intraperitoneally at time of infection to induce protracted infection. B cell transfers and treatments started after 2 weeks post-infection. KL25H BCR transgenic mice with a congenic CD45.1 marker were used as donors. The KL25H mice were obtained from Dr. Daniel Pinschewer (European Virus Archive), and contain a knock-in for a BCR heavy chain locus specific for LCMV WE-GP (1, 27). Note that the KL25H B cells from these transgenic mice must undergo pairing with endogenous light chains to become LCMV-specific. B cells were purified from spleen and bone marrow of transgenic KL25H mice, using a negative selection isolation kit (STEMCELL Technologies), and purity was confirmed to be >97%. 5x10^6^ KL25H B cells were injected into mice. SMARTA TCR transgenic mice (developed by Dr. Annette Oxenius (28) and deposited in Jackson laboratories, Strain #:030450) were used as donors. SMARTA CD4 T cells (CD45.1) were first MACS-purified from spleen of SMARTA mice, using a negative selection MACS isolation kit (STEMCELL Technologies). These naïve SMARTA cells were transferred into congenically distinct mice, which were then infected with LCMV Armstrong to generate a large pool of effector CD4 T cells. At day 7 post-infection, effector SMARTA CD4 T cells were MACS- and FACS-purified by negative selection from spleen and bone marrow, as shown previously (29), and 10^6^ cells were transferred into chronically infected mice with or without KL25 B cells.

### Quantification of viral titers

Viral load quantification was performed using Vero E6 cells, as described previously (21, 30). In brief, Vero E6 monolayers were plated onto 6-well plates, and after 24~48 hrs when they reached ~95% confluency, the media were removed and 200 μL of serial viral dilutions were added dropwise on top of the cells. Plates were rocked every 10 min in a 37°C, 5% CO_2_ incubator. After 1 hr, 200 μL of media was aspirated and the monolayers were gently overlaid with a 1:1 mixture of 2x 199 media (20% FBS, 2% Pen/Strep, 2% L-glutamine) and 1% agarose at 37°C. After 4 days, a second overlay was added, consisting of a 1:1 solution of 2x 199 media, 1% agarose, and 1:50 of neutral red. Overlay was removed on day 5 and plaques were counted. All mouse experiments were performed with approval of the Northwestern University Institutional Animal Care and Use Committee (IACUC).

### Neutralization assays

Serum samples were heat-inactivated at 45°C in water bath for 30 minutes. Mixture of serially diluted serum samples and the diluted virus were incubated for 60 minutes at 37°C in a CO_2_ incubator to allow the serum to neutralize the virus. After the incubation, 100 μL of the mixture of the serially diluted serum samples and virus were added onto a 96-well half-area plate with Vero E6 cells (2x10^4^ cells/well) and incubated overnight at 37°C in the CO_2_ incubator.

On the next day, the inoculum was removed, and the cells were fixed with 4% paraformaldehyde for 30 minutes at room temperature. The plates were washed with PBS twice and blocked for 1 hour at room temperature with the blocking buffer (3% BSA, 0.3% Triton X-100, 10% FBS in PBS). After the blocking step, rat anti-LCMV antibody (VL4) in blocking buffer were added, and incubated for 1 hour at room temperature. The plates were washed with PBS twice and incubated with donkey anti-rat IgG Alexa Fluor 488 in the blocking buffer for 40 minutes at room temperature. The plates were washed with PBS 3 times and 100 μL of PBS were added in each well. The plates were kept in 4°C and the foci were counted using an EVOS FL digital inverted microscope.

### Enzyme-linked immunosorbent assay

Virus-specific ELISAs were performed for quantification of antibody responses, similar to earlier studies (31–35). Briefly, 96-well MaxiSorp plates (Thermo Fisher Scientific) were coated with 100 μL/well of LCMV lysate diluted 1:10 in PBS for 48 hours. Plates were washed 3 times with wash buffer (PBS plus 0.5% Tween 20) followed by blocking with blocking solution (200 μL/well PBS plus 0.2% Tween 20 plus 10% FCS) for 2 hours. 5 μL of sera were added to 145 μL blocking solution in the first column of the plate, and 1:3 serial dilutions were performed for each sample followed by incubation for 90 minutes. Plates were washed 3 times with wash buffer, followed by addition of 100 μL/well HRP-conjugated goat anti–mouse IgG (SouthernBiotech), diluted 1:5000 in blocking solution. Plates were incubated for 90 minutes. After washing the plates 3 times with wash buffer, 100 μL/well SureBlue Substrate (SeraCare) was added for ~ 8 minutes. The reaction was stopped using 100 μL/well KPL TMB Stop Solution (SeraCare). Absorbance was measured at 450 nm using a Spectramax Plus 384 (Molecular Devices).

### Reagents, flow cytometry and antibodies for *in vitro* experiments

Single cell suspensions were obtained from PBMCs using Ficoll-Paque (GE Healthcare) gradient centrifugation or from tissues. For flow cytometry analysis, live cells were gated using Live/Dead fixable dead cell stain (Invitrogen). MHC class I tetramers were obtained from the NIH tetramer facility (Emory University). Anti-mouse flow cytometry antibodies were purchased from BD Pharmingen or Biolegend. Flow cytometry samples were acquired with a Becton Dickinson (BD) LSR Fortessa II or BD FACS Canto II and analyzed using FlowJo (Treestar).

### Statistical analysis

Statistical analyses were performed using the Mann Whitney test, unless noted otherwise in the figure legends. Data were analyzed using Prism software (Graphpad). Statistical significance was established at p ≤ 0.05.

## Data availability statement

The raw data supporting the conclusions of this article will be made available by the authors, without undue reservation.

## Ethics statement

The Mouse experiments were reviewed and approved by the Institutional Animal Care and Use Committee (IACUC) at Northwestern University (protocols IS00003258, IS00008785, IS00003324). All experiments were performed minimizing mouse distress in accordance with recommendations listed in the Guide for the Care and Use of Laboratory Animals of the NIH.

## Author contributions

PP-M wrote this article, and all other authors performed the experiments in this paper. All authors contributed to the article and approved the submitted version.

## Funding

This work was possible with a grant from the National Institute on Drug Abuse (NIDA, DP2DA051912) to PP-M.

## Acknowledgments

We thank Drs. Rafi Ahmed, Daniel Pinschewer, and Andreas Wieland for discussions. We also thank Dr. Mincheol Park for technical help.

## Conflict of interest

The authors declare that the research was conducted in the absence of any commercial or financial relationships that could be construed as a potential conflict of interest.

## Publisher’s note

All claims expressed in this article are solely those of the authors and do not necessarily represent those of their affiliated organizations, or those of the publisher, the editors and the reviewers. Any product that may be evaluated in this article, or claim that may be made by its manufacturer, is not guaranteed or endorsed by the publisher.

## Supplementary material

Supplementary Figure 1 Splenic germinal center B cell responses following co-transfer of virus-specific CD4 T cells (SMARTA) and B cells (KL25H). **(A)** Representative FACS plots showing the frequencies of KL25 B cells, isotype-switched B cells, and germinal center B cells in spleen. **(B)** Percentage of isotype-switched B cells in spleen. **(C)** Total number of germinal center (GC) B cells in spleen. Experiments were performed 2 times, n=3-5 mice per experiment. Error bars represent SEM. The p-values were calculated using Mann-Whitney test. ns, not significant.

Supplementary Figure 2 CD8 T cells following co-transfer of B cells and CD4 T cells. Summary of LCMV-specific (GP276-specific) CD8 T cells in blood. Experiments were performed 3 times, n=3-5 mice per experiment. Error bars represent SEM. The p-values were calculated using Mann-Whitney test. ns, not significant.

Supplementary Figure 3 Adoptive B cell therapy also improves viral control in a model of protracted infection caused by acute IFN-I blockade. **(A)** Experimental outline for evaluating the effect of B cell transfer during a protracted viral infection caused by IFN-I blockade. Mice received 500 μg of anti-IFNAR1 antibody (MAR1-5A3) 6 hours prior to LCMV Cl-13 WE-GP infection. Mice received 5x10^6^ KL25H B cells after 2 weeks post-infection. **(B)** Summary of viral control in sera. Data are from one representative experiment with, n=4-5 mice per experiment. Error bars represent SEM. The p-values were calculated using Mann-Whitney test. *P<0.05.
