# Supplementary figures and images for: Adoptive B cell therapy for chronic viral infection
Source: Front Immunol. 2022 Jul 26;13:908707. doi: 10.3389/fimmu.2022.908707 (PMC9361846; doi:10.3389/fimmu.2022.908707)

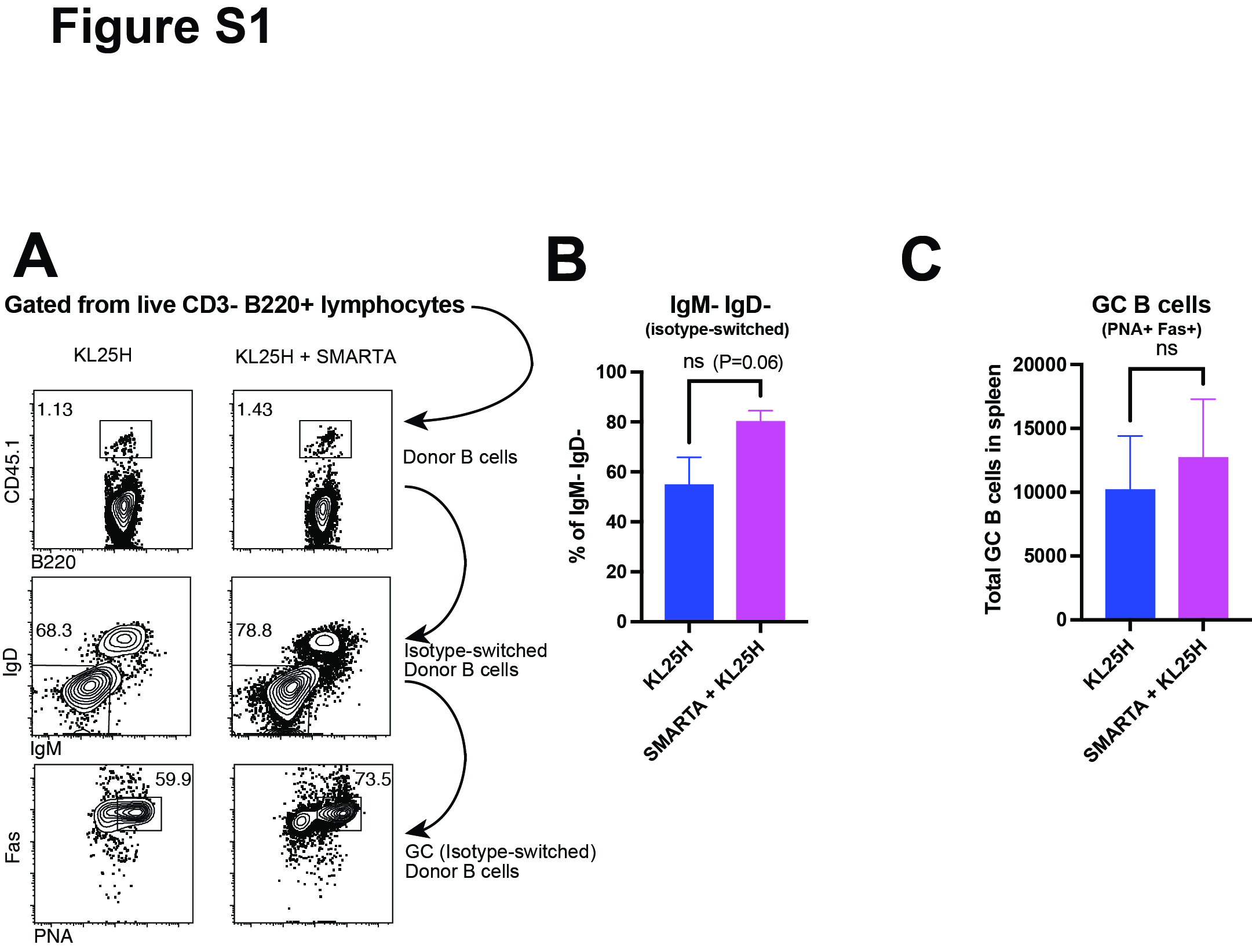

Supplement: Supplementary Figure 1 — Splenic germinal center B cell responses following co-transfer of virus-specific CD4 T cells (SMARTA) and B cells (KL25H). (A) Representative FACS plots showing the frequencies of KL25 B cells, isotype-switched B cells, and germinal center B cells in spleen. (B) Percentage of isotype-switched B cells in spleen. (C) Total number of germinal center (GC) B cells in spleen. Experiments were performed 2 times, n=3-5 mice per experiment. Error bars represent SEM. The p-values were calculated using Mann-Whitney test. ns, not significant. [file Image_1.jpg]

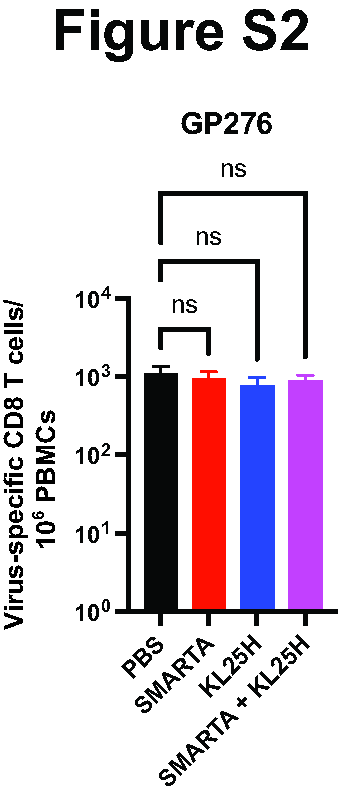

Supplement: Supplementary Figure 2 — CD8 T cells following co-transfer of B cells and CD4 T cells. Summary of LCMV-specific (GP276-specific) CD8 T cells in blood. Experiments were performed 3 times, n=3-5 mice per experiment. Error bars represent SEM. The p-values were calculated using Mann-Whitney test. ns, not significant. [file Image_2.tiff]

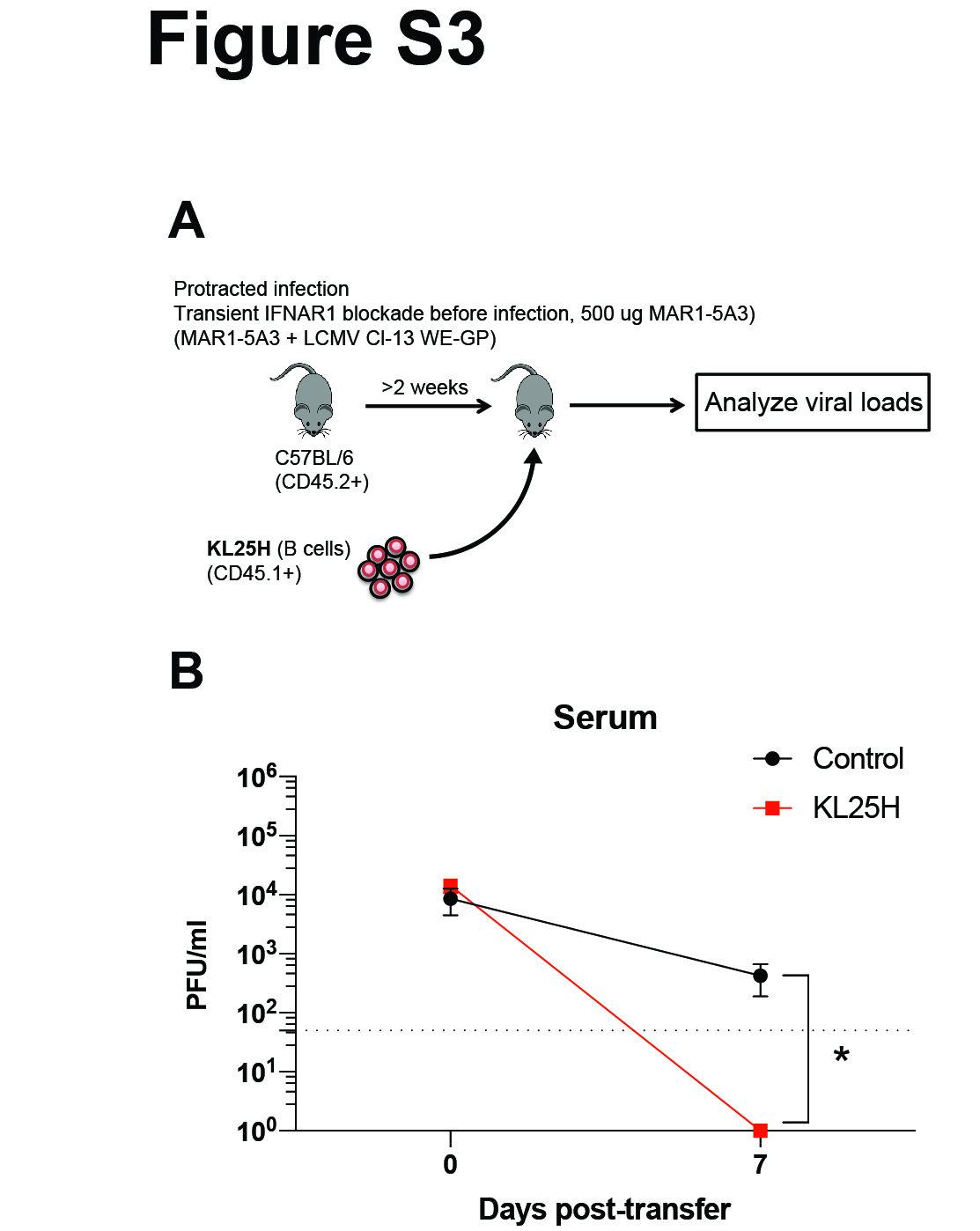

Supplement: Supplementary Figure 3 — Adoptive B cell therapy also improves viral control in a model of protracted infection caused by acute IFN-I blockade. (A) Experimental outline for evaluating the effect of B cell transfer during a protracted viral infection caused by IFN-I blockade. Mice received 500 μg of anti-IFNAR1 antibody (MAR1-5A3) 6 hours prior to LCMV Cl-13 WE-GP infection. Mice received 5x10^6^ KL25H B cells after 2 weeks post-infection. (B) Summary of viral control in sera. Data are from one representative experiment with, n=4-5 mice per experiment. Error bars represent SEM. The p-values were calculated using Mann-Whitney test. *P<0.05. [file Image_3.jpg]
